# Emergence of Avian Influenza A(H7N9) Virus Causing Severe Human Illness — China, February–April 2013

**Published:** 2013-05-10

**Authors:** China–US Collaborative Program on Emerging and Re-emerging Diseases, Chinese Center for Disease Control and Prevention and CDC, Beijing, China, US Dept of Agriculture. Div of Global Migration and Quarantine, National Center for Emerging and Zoonotic Infectious Diseases, Div of State and Local Readiness, Office of Public Health Preparedness and Response, Influenza Coordination Unit, Office of Infectious Diseases, Influenza Div, Immunization Svcs Div and Office of the Director, National Center for Immunization and Respiratory Diseases, CDC

On March 29, 2013, the Chinese Center for Disease Control and Prevention completed laboratory confirmation of three human infections with an avian influenza A(H7N9) virus not previously reported in humans (1). These infections were reported to the World Health Organization (WHO) on March 31, 2013, in accordance with International Health Regulations. The cases involved two adults in Shanghai and one in Anhui Province. All three patients had severe pneumonia, developed acute respiratory distress syndrome (ARDS), and died from their illness (2). The cases were not epidemiologically linked. The detection of these cases initiated a cascade of activities in China, including diagnostic test development, enhanced surveillance for new cases, and investigations to identify the source(s) of infection. No evidence of sustained human-to-human transmission has been found, and no human cases of H7N9 virus infection have been detected outside China, including the United States. This report summarizes recent findings and recommendations for preparing and responding to potential H7N9 cases in the United States. Clinicians should consider the diagnosis of avian influenza A(H7N9) virus infection in persons with acute respiratory illness and relevant exposure history and should contact their state health departments regarding specimen collection and facilitation of confirmatory testing.

## Epidemiologic Investigation

**FIGURE 1:**
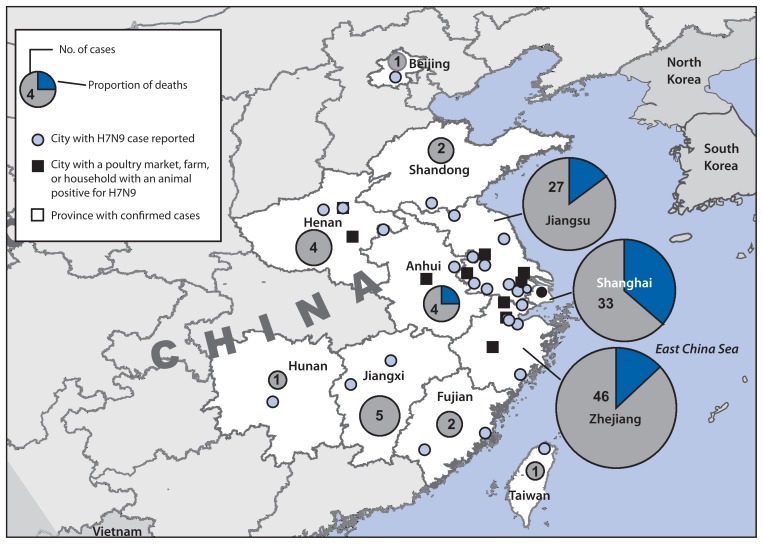
Location of confirmed cases of human infection (n = 126) with avian influenza A(H7N9) virus and deaths (n = 24) — China, February 19–April 29, 2013

**FIGURE 2:**
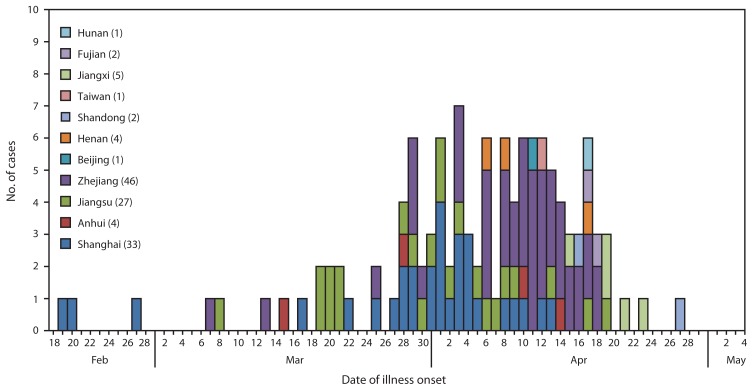
Number of confirmed cases of human infection with avian influenza A(H7N9) virus (N = 126), by date of onset of illness and province, municipality, or area — China, February 19–April 29, 2013

As of April 29, 2013, China had reported 126 confirmed H7N9 infections in humans, among whom 24 (19%) died (1). Cases have been confirmed in eight contiguous provinces in eastern China (Anhui, Fujian, Henan, Hunan, Jiangsu, Jiangxi, Shandong, and Zhejiang), two municipalities (Beijing and Shanghai), and Taiwan (Figure 1). Illness onset of confirmed cases occurred during February 19–April 29 (Figure 2). The source of the human infections remains under investigation. Almost all confirmed cases have been sporadic, with no epidemiologic link to other human cases, and are presumed to have resulted from exposure to infected birds (3,4). Among 82 confirmed cases for which exposure information is available, 63 (77%) involved reported exposure to live animals, primarily chickens (76%) and ducks (20%) (3). However, at least three family clusters of two or three confirmed cases have been reported where limited human-to-human transmission might have occurred (3).

The median age of patients with confirmed infection is 61 years (interquartile range: 48–74); 17 (21%) of the cases are among persons aged ≥75 years and 58 (71%) of the cases are among males. Only four cases have been confirmed among children; in addition, a specimen from one asymptomatic child was positive for H7N9 by real-time reverse transcription–polymerase chain reaction (rRT-PCR). Among the 71 cases for which complete data are available, 54 (76%) patients had at least one underlying health condition (3). Most of the confirmed cases involved severe respiratory illness. Of 82 confirmed cases for which data were available as of April 17, 81 (99%) required hospitalization (3). Among those patients hospitalized, 17 (21%) died of ARDS or multiorgan failure, 60 (74%) remained hospitalized, and only four (5%) had been discharged (3).

Chinese public health officials have investigated human contacts of patients with confirmed H7N9. In a detailed report of a follow-up investigation of 1,689 contacts of 82 infected persons, including health-care workers who cared for those patients, no transmission to close contacts of confirmed cases was reported, although investigations including serologic studies are ongoing (3). In addition, influenza surveillance systems in China have identified no sign of increased community transmission of this virus. Seasonal influenza A(pH1N1) and influenza B viruses continue to circulate among persons in areas where H7N9 cases have been detected, and the Chinese Centers for Disease Control and Prevention has reported that rates of influenza-like illness are consistent with expected seasonal levels.

CDC, along with state and local health departments, is continuing epidemiologic and laboratory surveillance for influenza in the United States. On April 5, 2013, CDC requested state and local health departments to initiate enhanced surveillance for H7N9 among symptomatic patients who had returned from China in the previous 10 days (5). As of April 29, 37 such travelers had been reported to CDC by 18 states. Among those 37 travelers, none were found to have infection with H7N9; seven had an infection with a seasonal influenza virus, one had rhinovirus, one had respiratory syncytial virus, and 28 were negative for influenza A and B. Among 31 cases with known patient age, seven travelers were aged <18 years, 13 were aged 18–64 years, and 11 were aged ≥65 years. Additionally, influenza activity in the United States is low and continues to decrease, with morbidity and mortality surveillance systems reporting activity below seasonal baseline levels. Although low numbers of influenza viruses are being detected, the majority in recent weeks have been influenza B.*

## Laboratory Investigation

As of April 30, 2013, Chinese investigators had posted 19 partial or complete genome sequences from avian influenza A(H7N9) viruses to a publicly available database at the Global Initiative on Sharing All Influenza Data (http://www.gisaid.org). Sequences are from viruses infecting 12 humans and five birds, and two are from viruses collected from the environment. These sequences indicate that all eight genes of the H7N9 virus are of avian origin, with the closest phylogenetic relatives from three Eurasian influenza virus lineages (H7N3 from domestic ducks, H7N9 from wild birds, and H9N2 from birds widely distributed throughout East Asia). In addition, genetic changes in the sequences are present that have been associated with adaptations leading to enhanced virus binding to and replication in mammalian respiratory cells and increased severity of infection (2,4,6).

**FIGURE 3:**
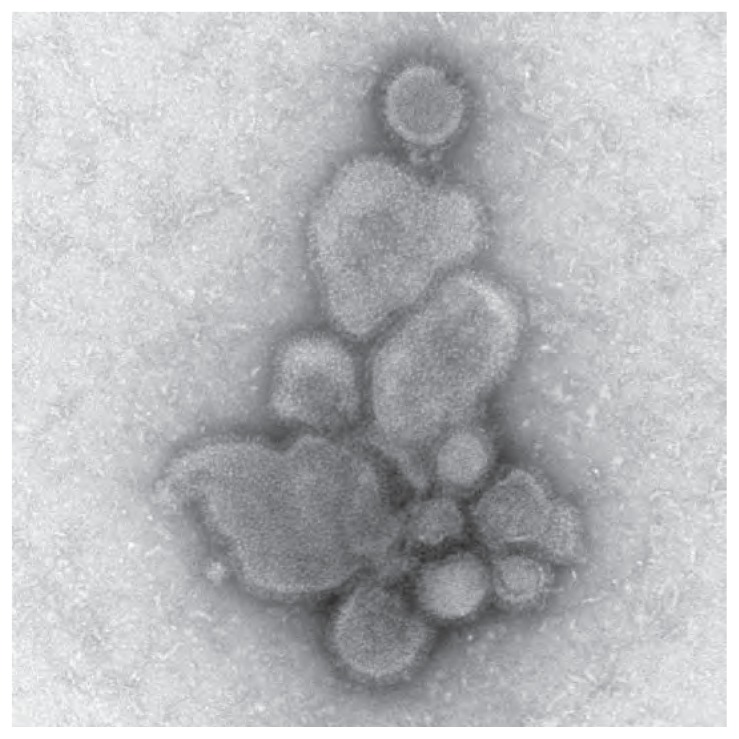
Electron micrograph image of influenza A/Anhui/1/2013 (H7N9), showing spherical virus particles characteristic of influenza virions — April 15, 2013 Photo/CDC

CDC’s Influenza Division Laboratory has received two H7N9 influenza viruses (A/Anhui/1/2013 and A/Shanghai/1/2013) from the WHO Collaborating Centre for Reference and Research on Influenza at the Chinese Center for Disease Control and Prevention (Figure 3). Full characterization of these viruses is ongoing; however, studies to date have shown robust viral replication in eggs, cell culture, and the respiratory tract of animal models (ferrets and mice). At higher inoculum doses (10^6^–10^4^ plaque forming units), the virus shows some lethality for BALB/c mice.

Laboratory testing of the A/Anhui/1/2013 virus isolate at the Chinese Center for Disease Control and Prevention, CDC, and other laboratories indicates that this virus is susceptible to oseltamivir and zanamivir, the two neuraminidase-inhibiting (NAI) antiviral drugs licensed in the United States for treatment of seasonal influenza. The genetic sequence of one of the publicly posted H7N9 viruses (A/Shanghai/1/2013) contains a known marker of NAI resistance (2). The clinical relevance of this genetic change is under investigation but it serves as a reminder that resistance to antiviral drugs can occur spontaneously through genetic mutations or emerge during antiviral treatment. The genetic sequences of all viruses tested showed a known marker of resistance to the adamantanes, indicating that, although these drugs (amantadine and rimantadine) are licensed for use in the United States, they should not be prescribed for patients with H7N9 virus infection.

Immediately after notification by Chinese health authorities of the H7N9 cases, CDC began development of a new H7 diagnostic test for use with the existing CDC influenza rRT-PCR kit. This test has been designed to diagnose infection with Eurasian H7 viruses, including the recently recognized China H7N9 and other representative H7 viruses from Southeast Asia and Bangladesh. On April 22, this new H7 test was cleared by the Food and Drug Administration for use as an in vitro diagnostic test under an Emergency Use Authorization, thus allowing distribution and use of the test in the United States. The CDC H7 rRT-PCR test is now available to all qualified U.S. public health and U.S. Department of Defense laboratories and WHO-recognized National Influenza Centers globally and can be ordered from the Influenza Reagent Resource (http://www.influenzareagentresource.org). Access to the CDC H7 rRT-PCR test protocol is available at http:/www.cdc.gov/flu/clsis. Guidance on appropriate biosafety levels for working with the virus and suspect clinical specimens is being developed.

## Animal Investigation and U.S. Animal Health Preparedness Activities

As of April 26, reports from the China Ministry of Agriculture indicate that 68,060 bird and environmental specimens have been tested, 46 (0.07%) were confirmed H7N9-positive by culture (7). The H7N9 virus has been confirmed in chickens, ducks, pigeons (feral and captive), and environmental samples in four of the eight provinces and in Shanghai municipality (Figure 1). As of April 17, approximately 4,150 swine and environmental samples from farms and slaughterhouses were reported to have been tested; all swine samples were negative.† The China Ministry of Agriculture is jointly engaged with the National Health and Family Planning Commission in conducting animal sampling to assist in ascertaining the extent of the animal reservoir of the H7N9 virus. Sampling of animals is concentrated in the provinces and cities where human cases have been reported. Poultry markets in Shanghai and other affected areas have been closed temporarily, and some markets might remain closed.

The U.S. Department of Agriculture (USDA) has set up a Situational Awareness Coordination Unit with a core team of subject matter experts and other USDA representatives, including the Animal and Plant Health Inspection Service (APHIS), the Agricultural Research Service (ARS), the Food Safety and Inspection Service, and the Foreign Agricultural Service. USDA and CDC are working collaboratively to understand the epidemiology of H7N9 infections among humans and animals in China. To date, no evidence of this strain of avian influenza A(H7N9) virus has been identified in animals in the United States. The U.S. government does not allow importation of live birds, poultry, and hatching eggs from countries affected with highly pathogenic avian influenza. The current U.S. surveillance program for avian influenza in commercial poultry actively tests for any form of avian influenza virus and would be expected to detect avian influenza A(H7N9) if it were introduced to the United States. A screening test for avian influenza is available from the National Animal Health Laboratory Network and the National Veterinary Services Laboratories (NVSL), which can be used together with confirmatory tests at NVSL to detect this strain of avian influenza A(H7N9) in poultry and wild bird samples.

APHIS is working with the U.S. Department of the Interior to prepare a pathway assessment, using current literature, to assess evidence for potential movement of Eurasian avian influenza viruses into North America via wild birds. USDA is conducting animal studies to characterize the virus pathogenicity and transmission properties of this virus in avian and swine species. Preliminary results from studies performed on poultry by ARS in high-containment laboratories indicate that chickens and quail are showing no signs of illness but are shedding avian influenza A(H7N9) virus in these studies (Southeast Poultry Research Laboratory, unpublished data; 2013). ARS also has completed a preliminary antigenic mapping study to help identify virus isolates that could be used to develop a vaccine for poultry if needed.

### Editorial Note

After recognition of the first human infections with avian influenza A(H7N9), Chinese public health officials and scientists rapidly reported information about identified cases and posted whole virus genome sequences for public access. During April, laboratory and surveillance efforts quickly characterized the virus, developed diagnostic tests, generated candidate vaccine viruses, identified cases and contacts, described clinical illness, evaluated animal sources of infection, and implemented control measures. Preliminary investigations of patients and close contacts have not revealed evidence of sustained human-to-human transmission, but limited nonsustained human-to-human H7N9 virus transmission could not be excluded in a few family clusters (3). Despite these efforts, many questions remain.

The epidemiology of H7N9 infections in humans so far reveals that most symptomatic patients are older (median age: 61 years), most are male (71%), and most had underlying medical conditions. In comparison, among the 45 avian influenza A(H5N1) cases reported from China during 2003–2013, the median patient age is 26 years (8). This difference in median age might represent actual differences in exposure or susceptibility to H7N9 virus infection and clinical illness, or preliminary H7N9 case identification approaches might be more likely to capture cases in older persons. Ongoing surveillance and case-control studies are needed to better understand the epidemiology of H7N9 virus infections, and to determine whether younger persons might be more mildly affected, and therefore less likely to be detected via surveillance.

What is already known on this topic?

Human infections with a new avian influenza A(H7N9) virus were first reported to the World Health Organization on March 31, 2013. Available information suggests that poultry is the source of infection in most cases. Although no evidence of sustained (ongoing) human-to-human spread of this virus has been identified; small family clusters have occurred where human-to-human spread cannot be conclusively ruled out.

What is added by this report?

By April 29, a total of 126 H7N9 human infections (including 24 deaths) had been confirmed. Although a number of travelers returning to the United States from affected areas of China have developed influenza-like symptoms and been tested for H7N9 infection, no cases have been detected in the United States. Laboratory and epidemiologic evidence suggest that this H7N9 virus is more easily transmitted from birds to humans than other avian influenza viruses. Candidate vaccine viruses are being evaluated and human clinical vaccine trials are forthcoming, but no decision has been made regarding a U.S. H7N9 vaccination program.

What are the implications for public health practice?

State and local health authorities are encouraged to review pandemic influenza preparedness plans to ensure response readiness. Clinicians in the United States should consider H7N9 virus infection in recent travelers from China who exhibit signs and symptoms consistent with influenza. Patients with H7N9 virus infection (laboratory-confirmed, probable, or under investigation) should receive antiviral treatment with oral oseltamivir or inhaled zanamivir as early as possible.

Available animal testing data and human case histories indicate that most human patients have poultry exposure; however, relatively few H7N9 virus–infected birds have been detected. During the month after recognition of H7N9, increasing numbers of infected humans have been identified in additional areas of eastern China, suggesting possible widespread occurrence of H7N9 virus in poultry. Enhanced surveillance in poultry and other birds in China is needed to better clarify the magnitude of H7N9 virus infection in birds and to better target control measures for preventing further transmission.

The emergence of this previously unknown avian influenza A(H7N9) virus as a cause of severe respiratory disease and death in humans raises numerous public health concerns. First, the virus has several genetic differences compared with other avian influenza A viruses. These genetic changes have been evaluated previously in ferret and mouse studies with other influenza A viruses, including highly pathogenic avian influenza A(H5N1) virus, and were associated with respiratory droplet transmission, increased binding of the virus to receptors on cells in the respiratory tract of mammals, increased virulence, and increased replication of virus (5). Epidemiologic investigations have not yielded conclusive evidence of sustained human-to-human H7N9 virus transmission; however, further adaptation of the virus in mammals might lead to more efficient and sustained transmission among humans. Second, human illness with H7N9 virus infection, characterized by lower respiratory tract disease with progression to ARDS and multiorgan failure, is significantly more severe than in previously reported infection with other H7 viruses. Over a 2-month period, 24 deaths (19% of cases) have occurred, compared with only one human death attributed to other subtypes of H7 virus reported previously. Third, H7N9-infected poultry are the likely source of infection in humans, but might not display illness symptoms. Consequently, efforts to detect infection in poultry and prevent virus transmission will be challenging for countries lacking a surveillance program for actively identifying low-pathogenicity avian influenza in poultry. In the United States, an active surveillance program is in place that routinely identifies low–pathogenicity viruses. If this newly recognized H7N9 is detected, public health and animal health officials should identify means for monitoring the spread of asymptomatic H7N9 virus infections in poultry and maintain vigilance for virus adaptation and early indications of potential human-to-human transmission.

Beginning in early April 2013, CDC and U.S. state and local health departments initiated enhanced surveillance for H7N9 virus infections in patients with a travel history to affected areas. A new CDC influenza rRT-PCR diagnostic test has been cleared by the Food and Drug Administration under an Emergency Use Authorization and is being distributed to public health laboratories to assist in evaluating these suspect cases. Clinicians should consider the possibility of H7N9 virus infection in patients with illness compatible with influenza who 1) have traveled within ≤10 days of illness onset to countries where avian influenza A(H7N9) virus infection recently has been detected in humans or animals, or 2) have had recent contact (within ≤10 days of illness onset) with a person confirmed to have infection with avian influenza A(H7N9) virus. Because of the potential severity of illness associated with avian influenza A(H7N9) virus infection, CDC recommends that all H7N9 patients (confirmed, probable, or under investigation for H7N9 infection) receive antiviral treatment with oseltamivir or zanamivir as early as possible. Treatment should be initiated even >48 hours after onset of illness. Guidance on testing, treatment, and infection control measures for H7N9 cases has been posted to the CDC H7N9 website (9).

On April 5, CDC posted a Travel Notice on the Traveler’s Health website informing travelers and U.S. citizens living in China of the current H7N9 cases in China and reminding them to practice good hand hygiene, follow food safety practices, and avoid contact with animals (10). CDC and WHO do not recommend restricting travel to China at this time. If travelers to China become ill with influenza signs or symptoms (e.g., fever, cough, or shortness of breath) during or after returning from their visit, they should seek medical treatment and inform their doctor about their recent travel. Travelers should continue to visit www.cdc.gov/travel or follow @CDCtravel on Twitter for up-to-date information about CDC’s travel recommendations.

Given the number and severity of human H7N9 illnesses in China, CDC and its partners are taking steps to develop a H7N9 candidate vaccine virus. Past serologic studies evaluating immune response to H7 subtypes of influenza viruses have shown no existing cross-reactive antibodies in human sera. In addition, CDC has activated its Emergency Operations Center to coordinate efforts. In the United States, planning for H7N9 vaccine clinical trials is under way. Although no decision has been made to initiate an H7N9 vaccination program in the United States, CDC recommends that local authorities and preparedness programs take time to review and update their pandemic influenza vaccine preparedness plans because it could take several months to ready a vaccination program, if one becomes necessary. CDC also recommends that public health agencies review their overall pandemic influenza plans to identify operational gaps and to ensure administrative readiness for an influenza pandemic. Continued collaboration between the human and animal health sectors is essential to better understand the epidemiology and ecology of H7N9 infections among humans and animals and target control measures for preventing further transmission.

## Footnotes

* Additional information available at http://www.cdc.gov/flu/weekly.

† Additional information available at http://www.chinacdc.cn.
